# A simulation environment for robot-assisted endovascular interventions

**DOI:** 10.1007/s11548-025-03458-2

**Published:** 2025-06-24

**Authors:** Matteo Pescio, Chenhao Li, Dennis Kundrat, Maura Casadio, Giulio Dagnino

**Affiliations:** 1 University of Twente, Enschede, The Netherlands; 2 Politecnico di Torino, Turin, Italy; 3 Università degli Studi di Genova, Genoa, Italy; 4 Università degli Studi di Torino, Turin, Italy; 5 Fraunhofer Research Institution for Individualized and Cell-Based Medical Engineering, Lübeck, Germany

**Keywords:** Endovascular robotic surgery, Surgical simulation, Model-based force and shape sensing, Digital twin

## Abstract

**Purpose:**

Cardiovascular diseases are the leading cause of mortality globally. Advances in interventional radiology and endovascular devices have made endovascular procedures effective alternatives to traditional open surgery, leading to their routine application in clinical practice. Within this framework, novel technologies, including robotic platforms and navigation software, have been developed to assist clinicians in executing endovascular interventions with improved dexterity, enhanced guidance, and superior clinical training, ultimately yielding better patient outcomes.

**Methods:**

This study aims to develop a model-based simulation environment within the SOFA framework, to enable shape and force sensing for endovascular robotic procedures. The vascular catheter was modeled using beam theory, and realistic interactions between the catheter and vascular models were established using the finite element method (FEM) with both linear elastic and nonlinear hyper-elastic models. Experiments measured contact forces and positional changes during catheter insertion, comparing anatomical deformations with simulation results.

**Results:**

Experimental tests validated the simulated force and displacement measurements. The catheter contact force showed an absolute error of 0.0371 N (30.45%). Catheter tip displacement averaged 3.1 mm, and the proximal segment’s Fréchet distance averaged 3.6 mm. For the anatomical model, the elastic FEM model performed best, with deformation measurement errors of 34%, 19%, and 59% across three different force scenarios.

**Conclusion:**

The results indicate that the integration of advanced physical modeling, realistic human–robot interactions, and enhanced computational capabilities will facilitate the development of innovative solutions, enabling clinicians to achieve greater accuracy and reliability in minimally invasive surgical (MIS) applications, particularly in endovascular interventions.

**Supplementary Information:**

The online version contains supplementary material available at 10.1007/s11548-025-03458-2.

## Introduction

Cardiovascular diseases (CVDs) are the leading cause of mortality globally, accounting for an estimated 17.9 million deaths each year [1]. CVDs encompass a range of peripheral vascular disorders affecting both small vessels and major arteries, including the aorta, such as aneurysms [2]. Specifically, abdominal aortic aneurysms (AAAs) are responsible for approximately 1.2% of all deaths among men aged 65–85 years. The incidence of AAA has increased over the past two decades, partly attributable to population aging, a rise in smoking prevalence, the implementation of screening programs, and advancements in diagnostic technologies [3].

While open surgery remains the standard treatment for aneurysm repair, it is associated with prolonged operative times, the necessity for general anesthesia, and extended hospital stays and recovery periods. However, advancements in interventional radiology and endovascular devices have led to the emergence of endovascular aneurysm repair (EVAR) as a viable alternative. The primary objective of EVAR is to deploy custom-made stent grafts that restore vessel geometry and normal blood flow patterns, thereby preventing further weakening of the vessel wall and reducing the risk of aortic rupture [4]. Pre-curved catheters are frequently utilized in endovascular procedures to navigate the vasculature and reach target sites for therapeutic or diagnostic interventions, such as stenting or ablation in cases of aneurysms [5].

In the realm of endovascular treatments, novel technologies including robotic platforms, navigation software, and simulation environments have been recently developed. These innovations aim to support clinicians by enhancing dexterity, providing improved guidance, and facilitating advanced clinical training, ultimately leading to improved clinical outcomes.

Research in the field of surgical robotics for endovascular procedures is actively pursued by both industrial and academic entities. Several commercial platforms are presently available, including Stereotaxis’ Genesis and Niobe (St. Louis, Missouri, USA), Robocath’s R-One+ (Rouen, France), and Catheter Precision’s Amigo RCS (Mount Olive, New Jersey, USA). In parallel with these commercial developments, emerging research trends such as MRI compatibility, human–robot interface and enhanced robotic autonomy have driven the creation of robotic research platforms [6], as well as novel imaging techniques [7–9]. These advancements aim to improve the precision and functionality of surgical robots in complex vascular environments [10].

Additionally, recent research has focused on the development of simulation environments tailored for endovascular procedures. These simulation platforms serve multiple purposes, including physician training [11, 12], integration of haptic feedback with robotic systems [13], and validation of machine learning algorithms for task automation [14–16]. Such innovations are critical for advancing the capabilities of surgical robots, enhancing training methodologies, and ensuring the reliability and efficiency of automated surgical tasks.

This study introduces a novel simulation environment for robot-assisted endovascular interventions, focusing on force and shape sensing capabilities within the SOFA framework [17]. The environment incorporates detailed geometric and mechanical modeling of a peripheral catheter and an abdominal aorta phantom with an aneurysm, emphasizing accurate deformation prediction under applied forces for integration with endovascular robotic platforms [6].

Model validation was conducted through two simulation tests. The first assessed contact forces during catheter insertion at a constant velocity, while the second evaluated displacements in the left iliac artery under a constant force field. These tests confirmed the simulation environment’s ability to replicate physical interactions observed in endovascular procedures, highlighting its potential as an effective tool for medical training and robotic system development.

## Simulation environment

This section presents the development of the model-based simulation scenario. Both the catheter and the phantom are developed after the evaluation of the geometrical and mechanical properties of the real hardware available in our laboratories.

### Peripheral catheter

**Fig. 1:**
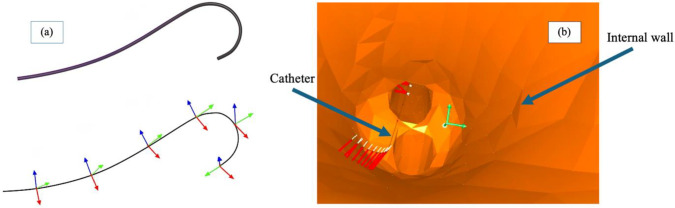
A peripheral catheter is modeled using beam elements, that were linked to recreate the shape. Each beam has its own reference system (**a**). The catheter is imported within the SOFA Framework to recreate a tool-insertion simulation scenario (**b**). Red lines represent the distance calculated by the contactDistance method when the distance between the instrument and the internal wall is less than the alarm distance set. The white lines indicate normals to the surface

A commercial Merit Medical Systems (South Jordan, UT, USA) Impress® Diagnostic Peripheral Catheter was modeled within SOFA using the Beam Adapter plugin [18], based on the one-dimensional finite element method (FEM). The catheter, 80 cm in length and 4 F (1.32 mm) in diameter, was geometrically represented in the simulation as a series of beam elements, each with two nodes possessing six degrees of freedom (Fig. 1a). This method decomposes motion into rigid body transformations and small deformations, enabling accurate simulation of catheter dynamics. The dynamics of the catheter model is governed by Newton’s second law of motion:

1 $$ M\left( x \right)\ddot{x} = F\left( t \right) - f\left( {x,\dot{x}} \right) + W\left( {x,\dot{x}} \right) $$

where $x$, $\dot{x}$, and $\ddot{x}$ represent the state (position, velocity, and acceleration) of the catheter model’s nodes; $M\left(x\right)$ and $f\left(x, \dot{x}\right)$ denote the inertia matrix and internal forces; $F(t)$ represents the external forces; and $W\left(x, \dot{x}\right)$ denotes the boundary conditions applied to the model.

In the simulation (Fig. 1b), boundary conditions are defined by interactions between the catheter and vessel walls. Contact detection is achieved using the *LocalMinDistance* method, which identifies potential contacts when the distance between collision elements falls below a specified alarm threshold (*alarmDistance*). If the distance decreases further, crossing a second threshold (*contactDistance*), a contact is established. After potential contact detection, the simulation determines actual contact occurrence and defines the corresponding response.

Unlike conventional tissue–tool interaction models that often neglect friction, this study incorporates a friction-inclusive contact model, as the phantom’s dry and textured material would render frictionless assumptions inaccurate. The model is based on Signorini’s contact law and Coulomb’s friction law [19]. We assigned the value of 0.62 to the friction coefficient $\upmu$. Furthermore, we opted for a model-tuning approach based on the available manufacturer specifications (length, diameter, material density) and an empirical calibration of beam model parameters (e.g., Young’s modulus) to evaluate which value was the best trade-off between accuracy and stability during the simulation for the catheter model. Nine catheter configurations were considered (Table 1) and qualitatively tested in a short benchmark insertion from the left iliac artery to the distal aorta. For each run, we visually inspected shape fidelity and contact stability while noting whether the frame-rate remained smooth for interactive use. Configurations with too few nodes were fast but visibly distorted; those with many nodes were realistic but sluggish. Model 6, with a Young’s modulus 600 GPa, a density of 1.55 g/mm^3^ and 50 nodes, was therefore selected as the best compromise.

**Table 1:** Nine different models of catheter setup parameters were tested using the Beam Adapter plugin

	Young’s modulus [GPa]	Material density [g/mm^3^]	Number of nodes
Model 1	200	1.55	50
Model 2	300	1.55	50
Model 3	600	1	50
Model 4	600	1.55	10
Model 5	600	1.55	25
Model 6*	600	1.55	50
Model 7	600	1.55	70
Model 8	600	4	50
Model 9	800	1.55	50

*The model 6 reports the configuration taken as a reference

### Abdominal aorta with aneurysm

**Fig. 2:**
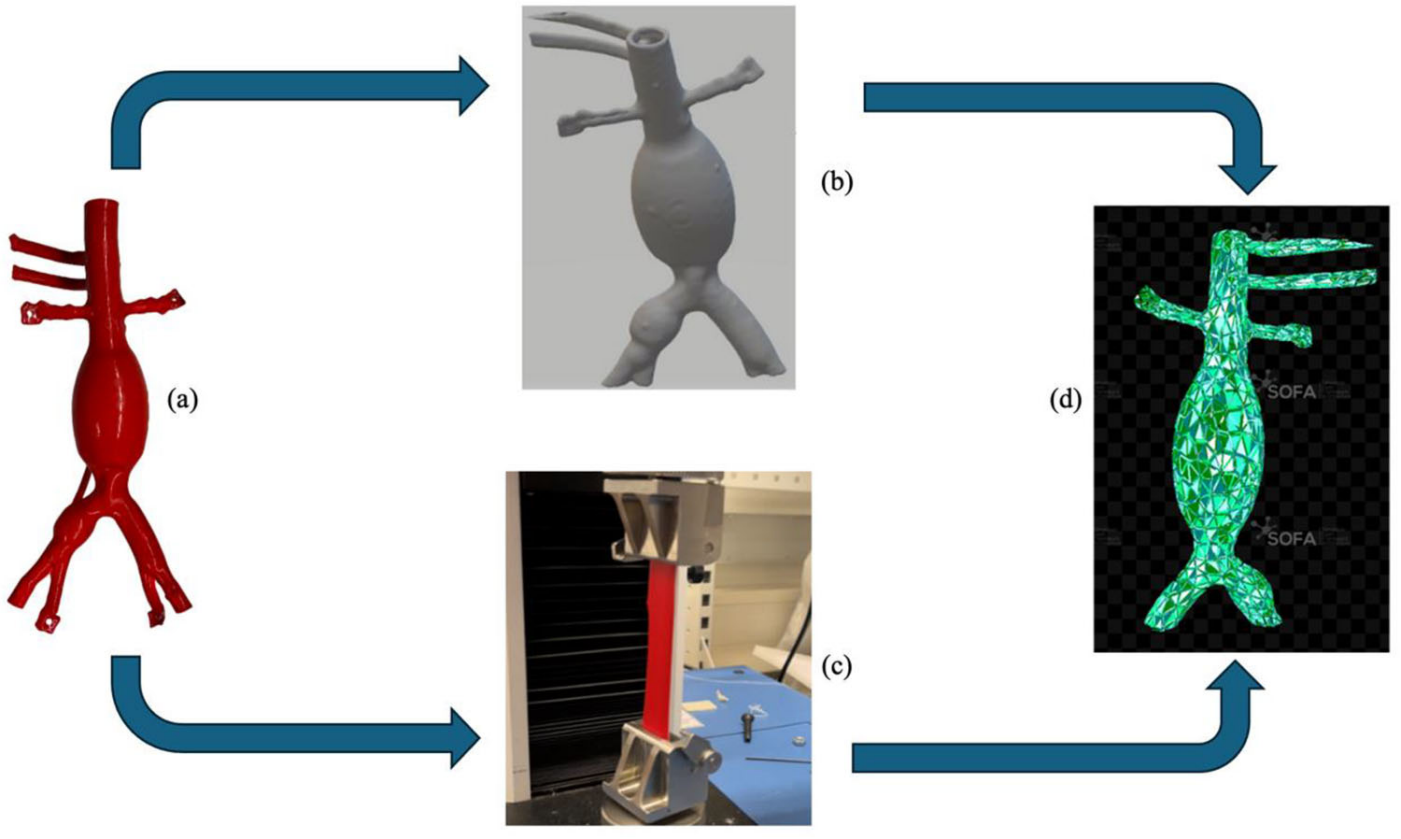
The workflow to develop our phantom digital twin: from a plastic phantom model (**a**), a tensile test (**c**) is performed on a material sample. A CT scan of the phantom is acquired (**b**), and its 3D model is segmented. The digital twin was developed in SOFA combining the mechanical with the geometric-anatomical properties (**d**)

The study involved the measurement of the mechanical properties of an abdominal aorta silicone phantom (Fig. 2a). To do so, a tensile test was conducted using a universal Instron 3343 compression and tension machine (Norwood, MA, USA), under the ASTM D412-16 standard (Fig. 2c). The machine, with a maximum capacity of 1 kN and a load accuracy of 0.5%, operated at a grip separation rate of 500 mm/min and a 10-Hz acquisition frequency. The material sample measured 70 mm in length, 32 mm in gauge length, 2 mm in thickness, and 27 mm in width, yielding a cross-sectional area of 54 mm^2^ (Fig. 2c). During the test, the sample was stretched to failure, and load-extension data were recorded. Stress–strain values were calculated to characterize the material’s tensile properties.

The hyperelastic models considered in this study include the neo-Hookean [20], Mooney–Rivlin [21], and Ogden [22] models, as they were previously studied to model the cardiovascular system [23]. The constitutive behavior of hyperelastic materials is derived from the Storage Energy Function (SEF) *W*, which is based on the three strain invariants ${I}_{1},{I}_{2},{I}_{3}$ [24]. The SEF represents the energy stored in the material per unit of reference volume (i.e., the volume in the initial configuration) as a function of the strain at a specific point within the material:

2 $$ W = f\left( {I_{1} ,I_{2} ,I_{3} } \right) $$

where *I*_1_, *I*_2_ and *I*_3_ are three invariants of Green deformation tensor [25] defined in terms of principal stretch ratios *λ*_1_, *λ*_2_ and *λ*_3_. They are expressed as:

3 $$ I_{1} = { }\lambda_{1}^{2} + { }\lambda_{2}^{2} + \lambda_{3}^{2} $$

4 $$ I_{2} = { }\lambda_{1}^{2} { }\lambda_{2}^{2} { + }\lambda_{2}^{2} { }\lambda_{3}^{2} { } + { }\lambda_{3}^{2} { }\lambda_{1}^{2} $$

5 $$ I_{3} = { }\lambda_{1}^{2} { }\lambda_{2}^{2} { }\lambda_{3}^{2} $$

Generally, hyperelastic materials are considered incompressible; hence, W is only a function of *I*_1_, *I*_2_ (i.e. *I*_3_ = 1). It means that the equation can be simplified as:

6 $$ W = W\left( {I_{1} - 3,I_{2} - 3} \right) $$

Furthermore, it was assumed that the material is also isotropic and nonlinear, following the approach used in [26] and that the tension is uniaxial on the material.

Consequently, the SEF of the neo-Hookean, Mooney–Rivlin and Ogden models is, respectively:

7 $$ W = C_{10} \left( {I_{1} - 3} \right) $$

8 $$ W = C_{10} \left( {I_{1} - 3} \right) + C_{01} \left( {I_{2} - 3} \right) $$

9 $$ W = { }\mathop \sum \limits_{i = 1}^{N} \frac{{2\mu_{i} }}{{\alpha_{i}^{2} }}(\lambda_{1}^{{\alpha_{i} }} + \lambda_{2}^{{\alpha_{i} }} + \lambda_{3}^{{\alpha_{i} }} - 3) $$

where *C*_01_, *C*_10_, *μ*_i_ and *α*_i_ are material constants. To calculate the constants, the constitutive equations were implemented in MATLAB’s curve fitting toolbox (Natick, MA, USA), fitting the stress–stretch curve obtained from tensile tests. The model fitting accuracy was evaluated using root-mean-square error (RMSE) and the coefficient of determination (*R*^2^), with all parameters reported at 95% confidence intervals (Table 2).

**Table 2:** Overview of the fitting analysis

Name	General model	Coefficients	R-square	RMSE
Elastic	$\sigma =E\varepsilon$	*E* = 0.46 MPa	0.99	0.07 MPa
Neo-Hookean	$\sigma =2\left({\lambda }^{2}-\frac{1}{\lambda }\right)\mu$	*μ* = 0.04 MPa	0.95	1.7 MPa
Mooney–Rivlin	$\sigma =2\left({\lambda }^{2}-\frac{1}{\lambda }\right)\left({C}_{10}+\frac{{C}_{01}}{\lambda }\right)$	*C*_01_ = 0.09 MPa *C*_10_ = 0.02 MPa	1.00	0.06 MPa
Ogden	$\sigma ={\mu }_{1}\left({\lambda }^{{\alpha }_{1}}-{\lambda }^{-\frac{1}{2}{\alpha }_{1}}\right)$	*α*_1_ = 1.4 *μ*_1_ = 0.20 MPa	1.00	0.04 MPa

Four different models are considered and compared. Ogden turns out to be the optimal one to fit the experimental data

CT scans of the phantom were segmented to generate a surface mesh representing the three-dimensional anatomy using a triangular mesh (Fig. 2b). To balance computational efficiency and accuracy, the number of nodes in the mesh was reduced, as conducting finite element analysis (FEA) on the original high-node mesh was computationally prohibitive.

The finite element method (FEM) provides a numerical approach to solving complex engineering problems by discretizing a continuous medium into elements interconnected by nodes [27]. Consequently, the global displacement is the sum of the displacements of the nodes.

Finite element (FE) models (Fig. 2d) were developed processing the phantom’s surface mesh in blender for decimation and smoothing. Also, only the left iliac artery region was considered for the simulation, being the region of interest (ROI) for our experimental validation (see Sect. "Experimental Validation"). Consequently, the ROI mesh had 477 vertices and 1000 faces, achieving a balance between fidelity and computational efficiency with a minimal distortion. The ROI model was then imported into Gmsh, where a tetrahedral volumetric mesh was generated by defining node positions and face connectivity. Using stress–stretch curve fits (Table 2), four three-dimensional FE models—elastic and hyperelastic—were created.

## Experimental validation

This section presents the experimental tests performed to assess the performance of the simulation scenario with respect to simple similar real cases.

### Catheter insertion

**Fig. 3:**
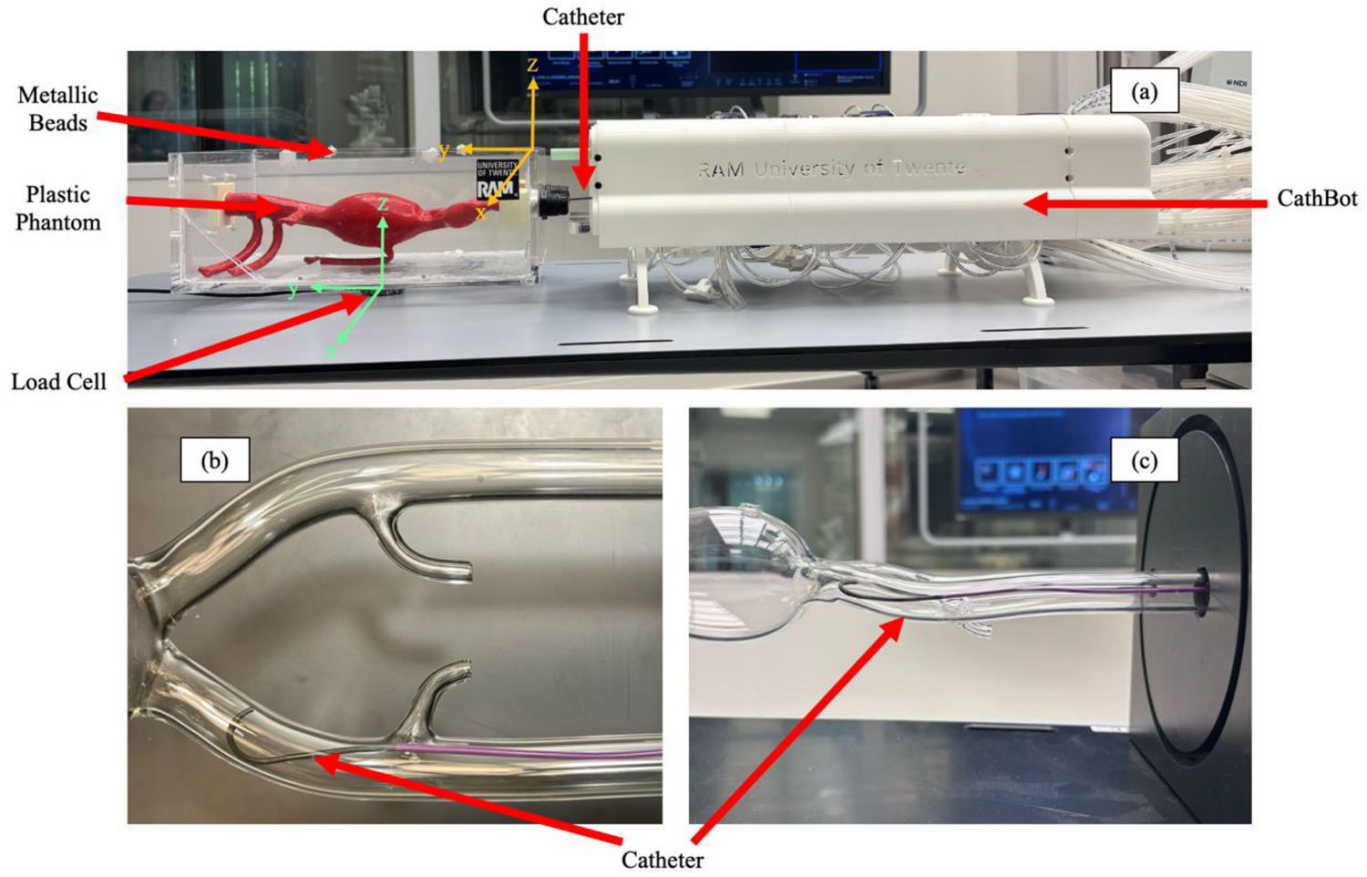
The experimental setup for the catheter insertion validation test. The test is intended to replicate the insertion phase of the catheter inside the left iliac artery using the CathBot robotic system (**a**). A bead-based registration is performed to track the catheter tip during the insertion, while an ATI mini 40 load cell measured the friction forces applied by the catheter. Top view (**b**) and side view (**c**) show that during the insertion, the catheter follows the central plane of the vessel

**Fig. 4:**
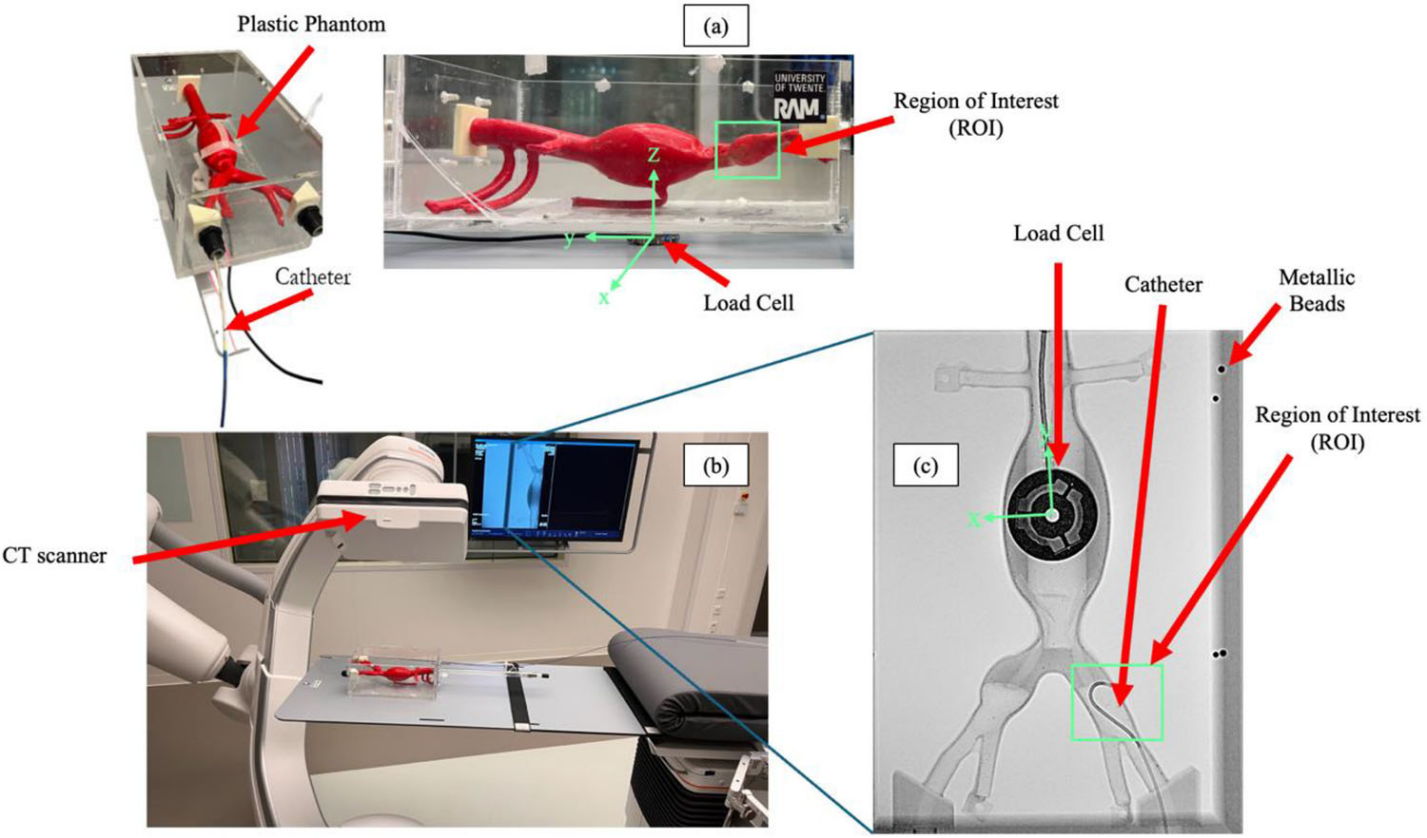
The experimental setup for the aortic deformation validation test. The test is meant to replicate a procedural error: the catheter hurts the internal wall of the iliac artery and causes a static deformation on the phantom (**a**). During the test the forces applied are calculated using the load cell, with respect to its reference system (**c**). Fluoroscopy images are acquired to register the deformations (**b**)

The first study conducted an experimental investigation to quantify the dynamic contact forces exerted between the catheter and the phantom during insertion into the left iliac artery extending to the abdominal aorta. Forces were measured using an ATI Mini-40 force/torque sensor (Apex, NC, USA). The sensor was positioned beneath the vascular phantom box (Fig. 3a). Catheter tip’s position was tracked using a C-arm Siemens Artis Pheno (Munich, Germany) (Fig. 4b), acquiring 2D images in the anterior–posterior (AP) plane. (Fig. 4c), under the assumption that the catheter approximately follows the central plane of the vessel (Fig. 3b), which is reasonable given the anatomical constraints imposed by the phantom geometry (Fig. 3c). We acquired volumetric scans of the vascular phantom before catheter insertion, with the catheter positioned at the entrance of the left iliac artery (Fig. 4a). This scan was used to determine the initial insertion point in both the physical and simulated environment. To enable accurate registration, the phantom box includes embedded metallic beads (Fig. 3a), which serve as fiducial markers. The catheter insertion was executed with the robotic platform CathBot [28] (Fig. 3a), enabling controlled insertion at constant speeds of 13 mm/s and 23 mm/s. Force and torque data were collected at a sampling rate of 1 kHz during the procedure.

The experimental procedure was replicated in simulation, where the catheter’s tip position was compared with simulated results. The metallic beads were also represented in the digital phantom, as we performed a bead-based registration [29], aligning the coordinates of the fiducial markers segmented from the C-arm scan with their corresponding positions in the simulation mode, to enable accurate spatial alignment between the real and virtual environments. The simulation employed the same insertion speeds of 13 mm/s and 23 mm/s as in the physical experiment for consistency.

A Bezier curve was used to fit the proximal part points, yielding a smoother curve for alignment with simulation outcomes.

### Aortic deformation

The second experimental test was designed to quantify the static forces exerted by a catheter on the inner surface of a vasculature model and to measure the resulting deformation, characterized by the displacement (in millimeters) of the anatomical region in contact with the catheter.

The procedure involved the insertion of the catheter into the phantom. In this instance, a deliberate static collision between the catheter and the inner vessel wall of the left iliac artery was induced to generate a measurable deformation. The ATI Mini-40 load cell, positioned beneath the phantom, measured the intensity of the force applied by the catheter on the region of interest. CT images of the phantom were acquired using the C-arm Siemens Artis Pheno both in the resting state and following deformation to assess the displacement of the anatomical structures (Fig. 4). The experiment was repeated three times, each iteration applying a qualitative incremental force that resulted in progressive deformation of the targeted anatomical region.

A total of four CT scans of the phantom were acquired during the experiment. The first scan was obtained before catheter insertion, illustrating the “resting” anatomy of the model. The subsequent three acquisitions corresponded to the deformations induced by the catheter during each of the three experimental repetitions.

The static force values measured during the experiments were subsequently applied in SOFA to the digital twin as a *ConstantForceField*. Corresponding strains in the anatomical region of interest were measured to evaluate the simulation’s accuracy.

## Results

This section presents the results from the two experimental tests and compares them with the corresponding simulation results. Initially, the outcomes of the catheter insertion test are discussed, followed by the results of the static deformation test. Refer to the Supplementary Material for the plots of the results.

### Vascular instrument model

Sensor data from catheter insertions (Fig. 3) showed that insertion speed minimally influenced force magnitude but affected the time to contact. Forces in the z direction, associated with gravity, were negligible in outcome determination, focusing the analysis on forces in the x and y axes at a speed of 13 mm/s. The data exhibited oscillations caused by speed instability and sensor noise, which were mitigated using outlier replacement, wavelet noise reduction, and a moving average filter leveraging the 1000 Hz sampling frequency. Comparisons between experimental data and simulations revealed that the directional limit device reduced forces during the pre-bent catheter tip insertion. Experimentally measured forces were lower than simulation predictions, with absolute errors of 0.0058 N (24.29%) in the x direction, 0.034 N (19.35%) in the y direction, and 0.0371 N (30.45%) for the total force.

Positional accuracy assessments during insertion reported an average Euclidean tip distance of 3.1 mm and a mean Fréchet distance of 3.6 mm for the proximal segment, confirming reasonable alignment between experimental and simulated data.

### Anatomical model

The stress–strain analysis of tensile test data (Fig. 2c) revealed rupture at 2.5 MPa stress and a strain of five times the initial length. Model fitting parameters and their accuracy were evaluated using root-mean-square error (RMSE) and the coefficient of determination (*R*^2^), with results summarized in Table 2. Among the models tested, the Ogden model demonstrated superior accuracy, outperforming the Mooney–Rivlin and elastic models. The neo-Hookean model exhibited the poorest fit, highlighting its limitations in representing the nonlinear, incompressible behavior of the silicone material in the phantom.

Force measurements were taken with an ATI Mini-40 load cell, and deformation was quantified from CT-based segmentations. Both analyses focused on changes in the left iliac artery caused by catheter insertion. Force data were classified as low, intermediate, or high, corresponding to 0.56 N, 1.10 N, and 1.63 N (± 0.01 N, load-cell resolution).

Mesh displacement analysis under *low, intermediate, and high* force conditions showed displacements of 3.34 mm, 4.61 mm, and 4.91 mm, respectively. Displacement measurements were anchored to anatomical landmarks and referenced against the nearest markers. Simulated displacements, presented in Table 3, were evaluated for each model under the three force conditions, ensuring consistency between experimental and simulation data through punctual measurements at specific mesh vertices. This methodology reinforces the fidelity of the simulated results relative to experimental observations.

**Table 3:** Comparison of experimental and simulated results

Model	Force [± 0.01 N]	Test displacement [± 0.01 mm]	Simulation displacement [± 0.01 mm]	Absolute error [± 0.01 mm]	Percentage error
Elastic	Low: 0.56	3.34	2.20	1.14	34%
	Intermediate: 1.1	4.61	5.24	0.63	14%
	High: 1.6	4.92	7.80	2.90	59%
Neo Hookean	Low: 0.56	3.34	0.85	2.49	74%
	Intermediate: 1.1	4.61	1.75	2.87	63%
	High: 1.6	4.92	2.72	2.19	45%
Mooney–Rivlin	Low: 0.56	3.34	0.39	2.95	88%
	Intermediate: 1.1	4.61	0.83	3.33	64%
	High: 1.6	4.92	1.28	3.91	80%
Ogden	Low: 0.56	3.34	0.31	3.03	90%
	Intermediate: 1.1	4.61	0.64	3.97	86%
	High: 1.6	4.92	1.00	3.91	80%

The absolute and percentage errors are calculated to compare the results. The elastic model turns out to be the most accurate during simulation scenarios

## Discussion

The primary forces are collision and friction with vessel walls. A direction limit device caused measured forces to be smaller than simulations and friction mainly affected the catheter entering the iliac arteries. During insertion, forces decreased or became negligible in the iliac arteries but surged upon contact with the abdominal aorta. Higher insertion speeds amplified this force increase, which then quickly reduced to sliding friction with the aorta wall. Force estimation accuracy was over 80% along the insertion direction and 70% in other directions, likely due to smaller deviations along the primary axis. The absolute error in force estimation (e.g., 30.45%) must be interpreted in light of the clinical application context. In endovascular interventions, the forces applied by catheters to vessel walls are typically low (in the range of 0.1–1.5 N) [30, 31]. Therefore, even a 30% error corresponds to a small absolute force (~ 0.03 N), which is unlikely to pose a significant clinical risk or affect surgical outcomes.

The Euclidean distance was employed to calculate the distance error of the catheter tip, while the Fréchet distance was calculated to assess the similarity between the proximal part curves. These metrics indicate a reasonable alignment between the simulation and experimental data, although discrepancies suggest areas for further refinement in the simulation model.

Catheter tip positional errors averaged 3.1 mm, higher than the 1.3 mm in [32] and lower than the 4.3 mm in [33]. Fréchet distance increased with insertion length, indicating greater similarity in proximal curves, potentially worsened by the lack of depth information. We consider the reported mean position error acceptable for this proof-of-concept validation, given that the catheter remains within the vessel lumen (approximately 8–10 mm in diameter), the primary goal was to validate trajectory and force response trends and the measured accuracy is consistent with or exceeds that reported in similar simulation-validation studies. Furthermore, the alignment of the catheter starting position within SOFA to match its physical location at the entrance of the iliac artery involved a manual step. Based on the image resolution (~ 0.3–0.5 mm) and the limited scope of manual intervention (catheter shape extraction and segmentation of the phantom were achieved using automated image processing techniques based on intensity thresholding and morphological operations), we estimate the cumulative measurement uncertainty to be approximately 1–1.5 mm, which is less than half of the reported 3.1-mm mean catheter tip error. As such, while measurement uncertainty contributes to the reported error, it does not dominate it. The reported value should be interpreted as a conservative upper bound on simulation accuracy.

Stress–strain curve was nearly linear, consistent with elastomer literature. The break point was around 550% strain, matching [34]. The elastic fit yielded a Young’s modulus of 0.46 MPa, comparable to the 0.53 MPa in [35]. Additionally, the Mooney–Rivlin *C*_01_ and *C*_10_ coefficients of 0.09 and 0.02 MPa are in the same measurement range of [35].

The elastic model performed best (Table 3) with percentage errors of 34%, 14%, and 59% for the three insertions, indicating acceptable reliability. Although the Ogden and Mooney–Rivlin models better fit the stress–strain, they had worse simulation results, with Ogden errors over 60%, likely due to linearizing hyperelastic models each timestep, affecting stability and real-time computation. Consequently, the elastic model is the optimal choice for simulating catheter interactions with the plastic phantom, effectively balancing accuracy and computational performance.

In the end, comparative analysis of simulation and experimental data highlights the platform’s efficacy in training medical personnel when supported by accurate biomechanical modeling.

We acknowledge that the mechanical fidelity of the phantom to real human vessels is a limitation, and we chose to study the application of isotropic hyperelastic models to fit the mechanical properties of the phantom. As future developments, we plan to include anisotropic hyperelastic models to better simulate the mechanical behavior of human blood vessels.

In addition, future developments will improve the fidelity of the simulation, to provide an advanced training environment for artificial intelligence models and enhance robotic surgical autonomy. Also, we consider to include imaging-derived patient-specific data, dynamic blood flow modeling, and deformation induced by cardio-respiratory activity, further improving the simulation’s realism and clinical applicability.

## Supplementary Information

Below is the link to the electronic supplementary material.

Supplementary file1 (DOCX 2244 KB)
