# Supplementary material for: A simulation environment for robot-assisted endovascular interventions
Source: Int J Comput Assist Radiol Surg. 2025 Jun 24;20(11):2259–67. doi: 10.1007/s11548-025-03458-2 (PMC12575544; doi:10.1007/s11548-025-03458-2)
Supplement: Supplementary file 1 — Supplementary file1 (DOCX 2244 KB) [file 11548_2025_3458_MOESM1_ESM.docx]

# **Supplementary Material**

1. **Supplementary figures: Plots with results**


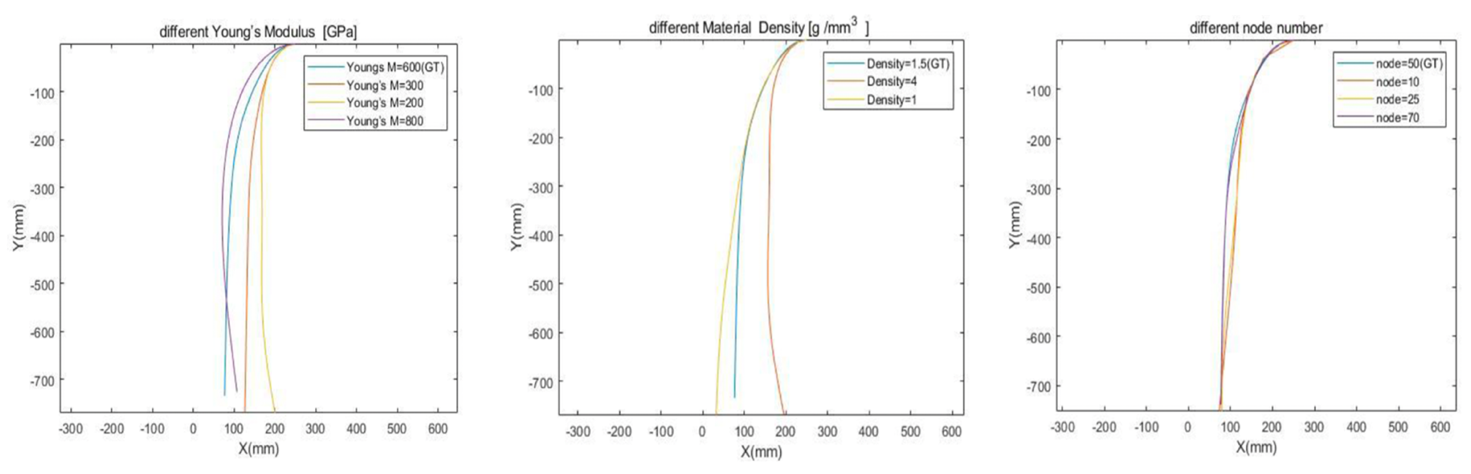


**Figure 1:** Catheter model shape configuration according to different Young’s Modulus (a), Material Density (b) and number of nodes (c).


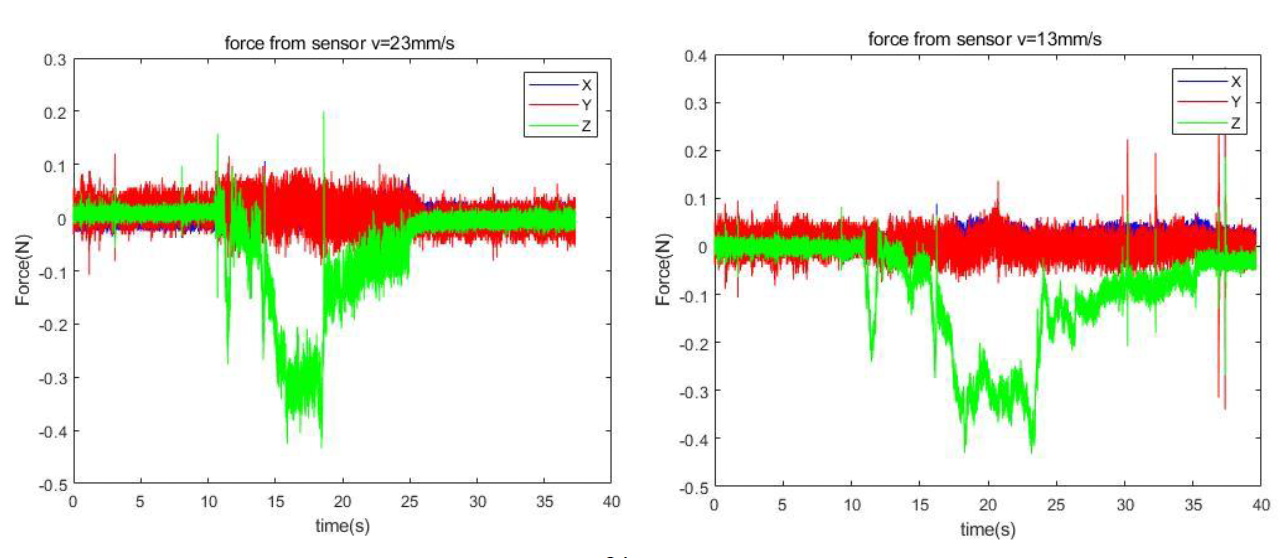


**Figure 2:** Profiles of the variation of the forces recorded with the ATI cell during the experiments, with a constant insertion speed of 23 mm/s (a) and 13 mm/s (b) respectively.


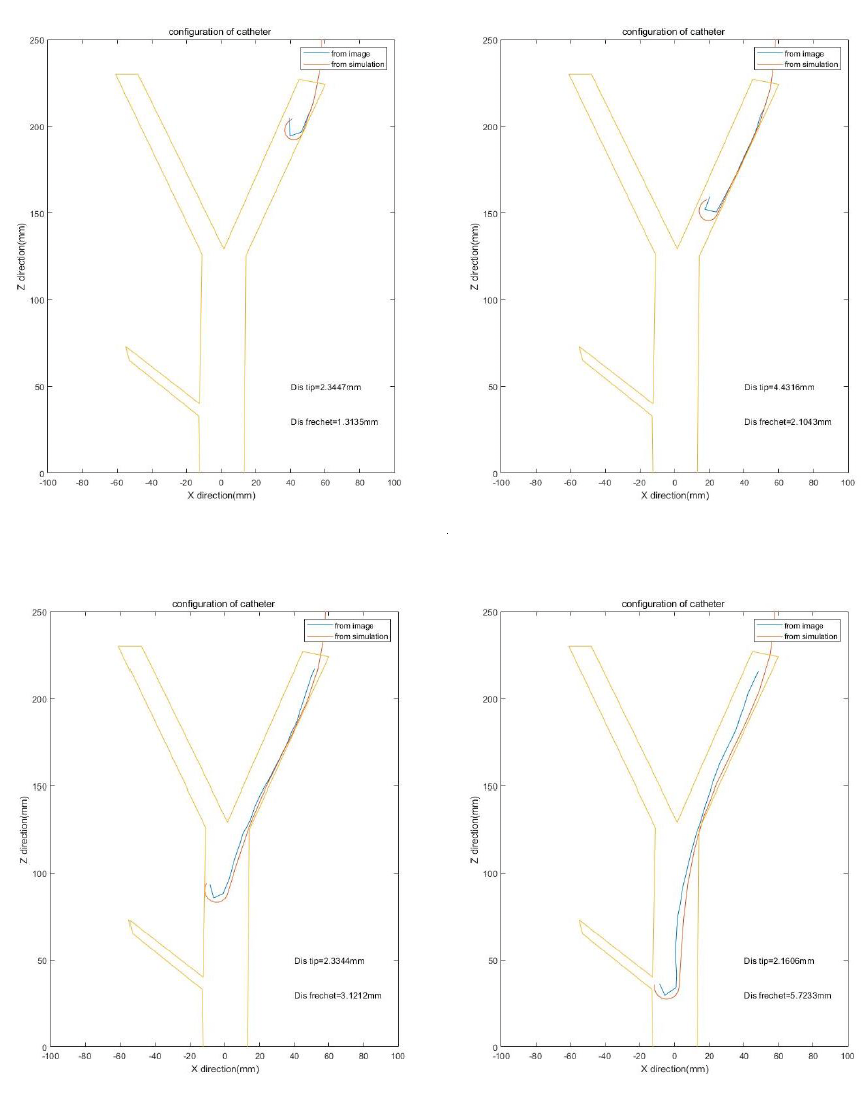


**Figure 3:** Evolution of the catheter movement (time interval = 4s) within the phantom, both from simulation scenario (orange line) and real experiment (blue line).


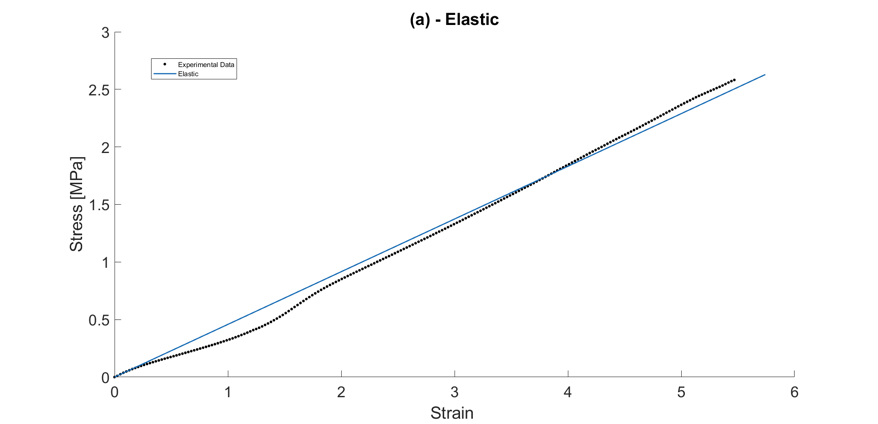

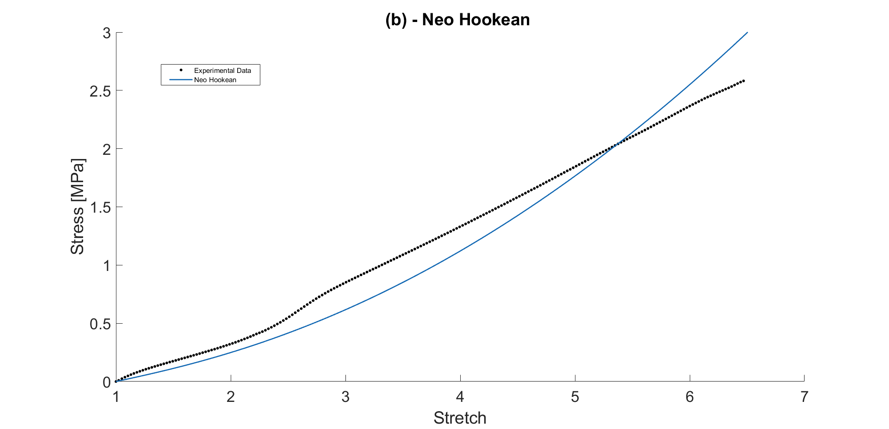


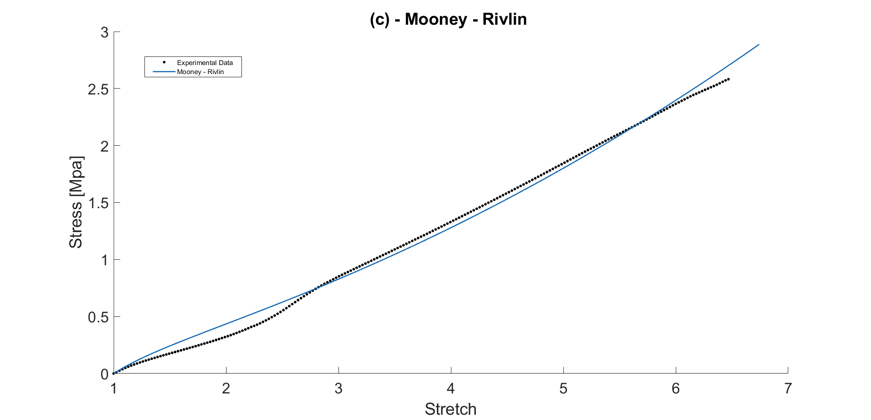

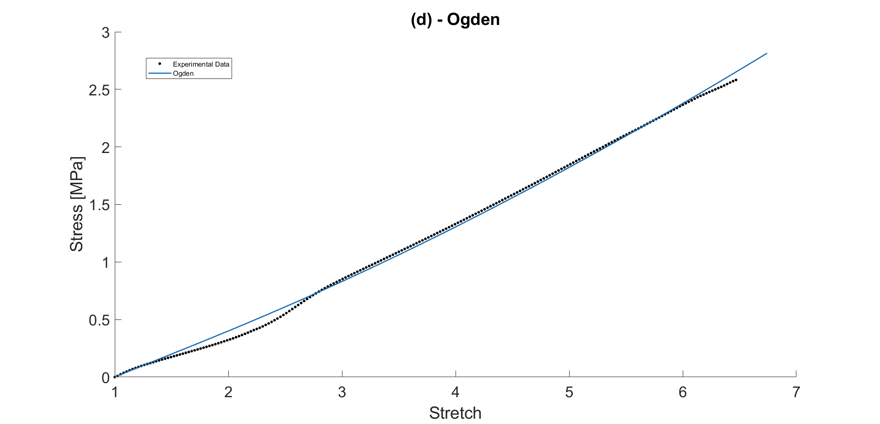


**Figure 4:** Plots of the model equations interpolated with the experimental data. The black dotted curve represents the experimental values of stress [MPa]/strain (or stress [MPa]/stretch in the case of hyperelastic materials) obtained by the Instron machine. The curves in blue represent the equations of the interpolating models, respectively the Elastic (a), Neo Hookean (b), Mooney-Rivlin(c), Ogden(d).


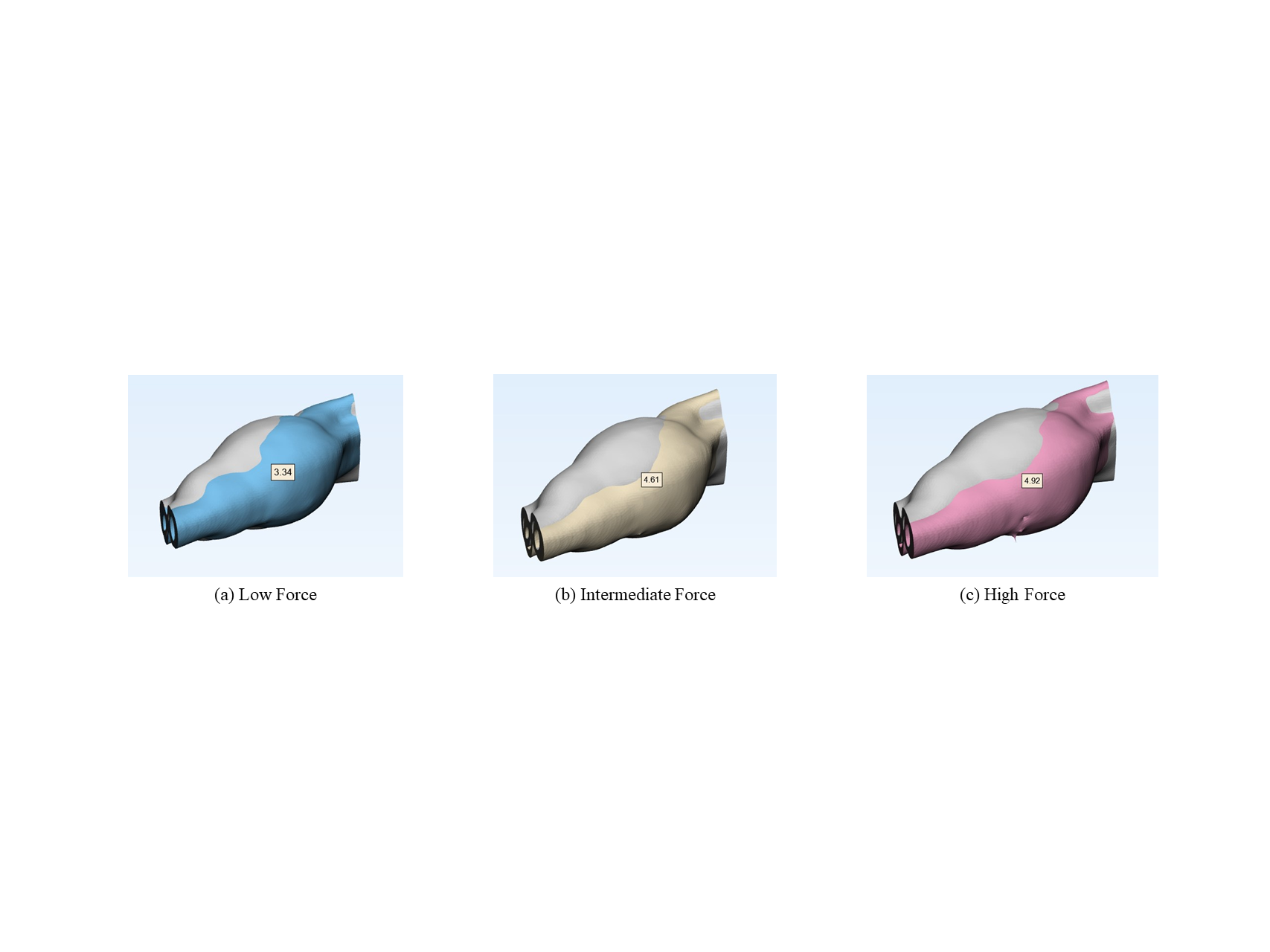


**Figure 5:** Deformations of the iliac artery according to (a) Low, (b) Intermediate, (c) High forces.


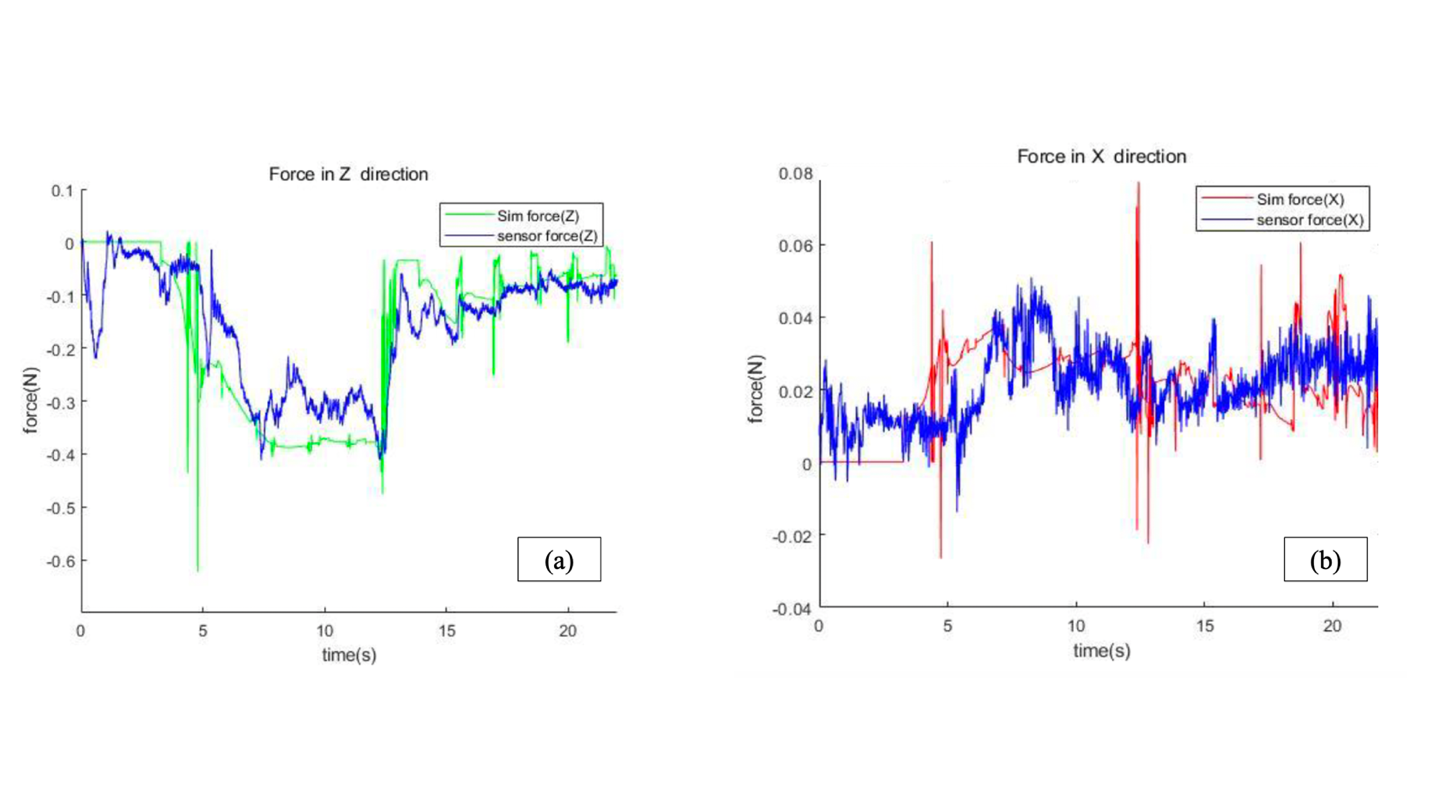


**Figure 6:** Comparison of force values along z (a) and x (b) directions both from the simulation scenario (red line) and from the real experiment (blue line)
